# Some Remarks on Conservative Gynecology

**Published:** 1902-04

**Authors:** E. C. Davis

**Affiliations:** Atlanta, Ga.


### SOME REMARKS ON CONSERVATIVE GYNECOLOGY.*

By E. C. DAVIS, M.D.,

ATLANTA, GA.

The subject selected for this evening's consideration is so broad and comprehensive that the limited time for its consideration but permits the skimming of the surface, and only allows us to point out some of its most interesting phases.

The meaning of the word conservative, as its etymology indicates, gives it a rather broad field, viz.: Con—together, and servo—to keep; that is, to keep together, to preserve. It has, however, been defined as opposed to new developments or to progress in any channel. This meaning I wish to entirely eliminate from the views entertained in the consideration of our subject. It should rather be applied to the conservation or restoration of the health of the patient by the removal only of such organs or tissues as are pathologic and causing the impairment of the patient's health.

Schafer and several other eminent physiologists, in their investigations of internal secretions, have caused the medical profession to consider seriously the conservation of all tissues, especially those of organic structure, for little know we what ultimate effects may follow the deprivation from the system of many of these so-called internal secretions, which we now know are produced by organs that are ductless, as well as those with ducts, and other known secretions.

Many of the profound nervous phenomena, following many of

* Paper read before meeting of Presbyterian Hospital Staff.

the operations, may, in a measure, be explained on the basis of an absence of some of these secretions. A diseased organ or tissue, on the other hand, may be producing products which impair the health of the patient, and in such cases the correction by judicious treatment, or the removal of these tissues, constitutes true conservatism. Again, complete or thorough surgery is often the most conservative, as its results justify the procedures, and we should be trained to discernment between conditions requiring extensive operative procedures and those requiring but little.

Conservatism should antecede operative procedures, and, in a measure, place the young woman in such a condition as to render operative measures unnecessary. Many young women are brought under the care of the gynecologist through ignorance or negligence of parents. Girls are allowed to reach puberty without information as to the menstrual function, and absolutely devoid of information as to how they should conduct themselves during this critical period of their histories. They are even allowed to enter into marital relations without proper information. Mothers can instruct their daughters in such a way as not to appear coarse or vulgar, and they owe that duty to their girls. Styles of dress play an important roll in this production of diseases of the generative organs. At the present time the style requires the well-dressed woman to wear a brace or support which causes her to bend forward away from the natural angle, and to accomplish this, a small pad is worn over the abdominal region pressing firmly in the direction of the fundus uteri. This, if persisted in, will conduce to displacements and cause the gynecologists to be busy for years to come. Again, too violent exercise so often leads to displaced or prolapsed ovaries and displacements of uterus. If we fail in these measures of prevention, then our attention is directed to a correction of the conditions.

In the early history of gynecological surgery the tendency was rather towards too frequent sacrifice of organs which might have been saved. I am quite sure that ovaries and tubes have been removed needlessly, and I very much fear lest many brilliant hysterectomies have been more beneficial to the operator's statistics than the patient's subsequent health.

Tears in cervix and perineum have been overlooked and neglected until they lead to more serious conditions of uterus, tubes or ovaries. True conservatism would tend to restore the parts to as nearly the normal condition as possible, hence these tears should receive early and complete repair. Within the past few months there has been a distinctive tendency on the part of some of the leading gynecologists to send the pendulum too far in the opposite direction, causing them often to leave tissues, which should be removed, and necessitating subsequent operations before relief is obtained. In making this discrimination, viz., to determine when a tissue or organ should be removed, we must bring to bear that strong and invaluable sense, commonly called practical sense, largely inherited, but susceptible of marked cultivation from extensive experience.

One operation which has only to be mentioned to be condemned, is double salpingo—oophorectomy for dysmenorrhea—in which the ovaries and tubes are in healthy condition. This has been quite a common operation—happily is not considered at the present time—as we are beginning to look more carefully on causes and endeavor to remove them without the sacrifice of useful organs. Many a woman with marked displacement of her uterus has been subjected to this operation. Again, I am constrained to believe that some women with marked nervous diseases, originating from causes other than pelvic disease, have been required to make this sacrifice.

On the other hand, how often does the gynecologist leave one ovary and later have this to remove before relieving his patient, the ovary appearing healthy on inspection. The great Lawson Tait advocated always the removal of both ovaries if one was diseased, as he stated that the one left almost invariably became diseased and would require removal. My own experience does not altogether corroborate this teaching, as occasionally an ovary may be left with benefit.

Another conservative operation, which occasionally deserves commendation, is the incision and drainage of small cysts in ovarian tissue and the breaking up of slight adhesions around tubes, due to slight trauma, without the sacrifice of the organs. If these little cysts be distributed extensively throughout the ovarian tissue, relief only follows the extirpation of the ovary.

Again, with suppurative conditions of tubes and ovaries and a septic metritis, conservatism would require the removal of the uterus, as well as its adnexæ, and a failure to remove this, as is often the case, leaves the patient with a septic focus which she carries constantly with her, producing a constant luccorrheal discharge, pains in the region of its location, and the patient is but little better off than before submitting to the grave operation.

Vaginal drainage, which has been called unsurgical, in many cases has saved the lives of women and rendered more extensive operations either unnecessary or simplified them greatly when they became necessary at some later date. This draining away of pus from the pelvis, removing, cleansing and asisting nature in the elimination of the toxins is a natural act and deserves commendation. This, too, requires much judgment and experience to keep from doing more harm than good, as it would be so easy to disseminate this purulent material throughout the entire abdominal cavity.

How often have we seen patients who have passed through operation after operation without any apparent relief for their symptoms! Then, too, have we observed many whose nervous symptoms have apparently been aggravated by precipitating the menopause upon a woman with an already overstrained nervous system. In some cases these symptoms have been so much intensified as to apparently lead to insanity or a profound form of hysteria. Cases of this kind are usually of mistaken diagnosis and belong in the first instance to the neurologist rather than the gynecologist. Such conditions so often lead to discouragement when viewed by the beginner and reflect greatly against our profession when the laity take cognizance of the results.

There is another condition which has often come under my observation, viz.: the advice of physicians to patients to have their uteri curetted, without more carefully examining the adnexæ; in fact, several cases have been seen by me in which this advice has been given the patients when suffering from suppurative diseases of tubes and ovaries, and the traction upon the uterus, in the performance of this operation, might rupture the tube and liberate the purulent material, producing a violent peritonitis. Just here I would like to state that the use of the curette is getting so common that it is often used injudiciously, and I would like to state that it is capable of much abuse and may prove a very dangerous instrument. Many a uterus has been perforated by this instrument and only the autopsy revealed its damaging results. I would not have you understand that I condemn the use of the curette, for such is not my intention, as I use it frequently and with much benefit, but I merely wish to caution against its careless or reckless abuse.

Fearing lest my remarks grow tiresome, I will mention what I consider some of the means of adhering to true conservatism:

1st. Make a careful diagnosis of the condition of your patient, and if you fail to find any pathologic condition, say so. Correct all displacements before serious operations are advised, when these are causing the discomfiture.

2d. Repair all lacerations before they lead to diseases of pelvic organs.

3d. Be extremely careful to determine the condition of uterus and adnexa before undertaking correction of displacements of uterus or plastic operations in this locality.

4th. Diagnose and operate early and thoroughly when malignant disease is detected in generative organs.

5th. Make careful examinations of urine and blood before major operations, if possible.

6th. Remember that women have other organs besides those of generation, and they, too, are subject to diseased conditions.

